# Whole exome sequencing confirms the clinical diagnosis of Marfan syndrome combined with X-linked hypophosphatemia

**DOI:** 10.1186/s12967-015-0534-9

**Published:** 2015-06-04

**Authors:** Xunlun Sheng, Xue Chen, Bo Lei, Rui Chen, Hui Wang, Fangxia Zhang, Weining Rong, Ruoshui Ha, Yani Liu, Feng Zhao, Peizeng Yang, Chen Zhao

**Affiliations:** Department of Ophthalmology, Ningxia Eye Hospital, Ningxia People’s Hospital, Ningxia, China; State Key Laboratory of Reproductive Medicine, Department of Ophthalmology, The First Affiliated Hospital of Nanjing Medical University, Nanjing, China; Chongqing Key Laboratory of Ophthalmology, Department of Ophthalmology, Chongqing Eye Institute, The First Affiliated Hospital of Chongqing Medical University, Chongqing, China; Human Genome Sequencing Center, Baylor College of Medicine, Houston, TX USA; Department of Radiology, Ningxia Eye Hospital, Ningxia People’s Hospital, Ningxia, China; Department of Cardiology and Surgery, Tianjin Chest Hospital, Tianjin Medical University, Tianjin, China

## Abstract

**Background:**

To determine the genetic lesions and to modify the clinical diagnosis for a Chinese family with significant intrafamilial phenotypic diversities and unusual presentations.

**Methods:**

Three affected patients and the asymptomatic father were included and received comprehensive systemic examinations. Whole exome sequencing (WES) was performed for mutation detection. Structural modeling test was applied to analyze the potential structural changes caused by the missense substitution.

**Results:**

The proband showed a wide spectrum of systemic anomalies, including bilateral ectopia lentis, atrial septal defect, ventricular septal defect, widening of tibial metaphysis with medial bowing, and dolichostenomelia in digits, while her mother and elder brother only demonstrated similar skeletal changes. A recurrent mutation, *PHEX* p.R291*, was found in all patients, while a *de novo* mutation, *FBN1* p.C792F, was only detected in the proband. The *FBN1* substitution was also predicted to cause significant conformational change in fibrillin-1 protein, thus changing its physical and biological properties.

**Conclusions:**

Taken together, we finalized the diagnosis for this family as X-linked hypophosphatemia (XLH), and diagnosed this girl as Marfan syndrome combined with XLH, and congenital heart disease. Our study also emphasizes the importance of WES in assisting the clinical diagnosis for complicated cases when the original diagnoses are challenged.

**Electronic supplementary material:**

The online version of this article (doi:10.1186/s12967-015-0534-9) contains supplementary material, which is available to authorized users.

## Background

Marfan syndrome (MFS; MIM 154700), characterized by complicated manifestations in multiple organ systems with high degrees of clinical diversity, is one of the most common autosomal dominant inherited connective tissue diseases with a prevalence of 1 in 5,000 [1]. Cardinal MFS features involve the ocular, skeletal, and cardiovascular systems, of which ectopia lentis and aortic aneurysm are given special clinical significance [2]. Excessive liner growth of the long bones and joint laxity are hallmarks for the skeletal systems. By far, mutations in two genes have been implicated in the etiology of MFS, including the fibrillin 1 (*FBN1*; MIM 134797) gene and the transforming growth factor beta receptor II (*TGFBR2*; MIM 190182) gene. Most MFS cases are caused by *FBN1* mutations [3], whereas the *TGFBR2* mutation has only been found correlated with MFS in a French family [4]. The diagnosis of MFS is usually based on both clinical signs and genetic findings [2].

Familial hypophosphatemic rickets (FHR) can be transmitted via all three types of Mendelian inheritance pattern. Autosomal dominant hypophosphatemic rickets (ADHR; MIM 193100) usually correlates with mutations in the fibroblast growth factor 23 (*FGF23*; MIM 605380) gene [5], whereas mutations in two other genes are implicated in causing the autosomal recessive form, including the dentin matrix acidic phosphoprotein 1 (*DMP1*; MIM 600980) gene associated with autosomal recessive hypophosphatemic rickets-1 (ARHR1; MIM 241520) [6], and the ectonucleotide pyrophosphatase/phosphodiesterase 1 (*ENPP1*; MIM 173335) gene related to autosomal recessive hypophosphatemic rickets-2 (ARHR2; MIM 613312) [7]. X-linked hypophosphatemia (XLH; MIM 307800), presenting a prevalence of 1 in 20,000, is the most common form of familial hypophosphatemic rickets (FHR), which is a dominant disorder biochemically featured by hypophosphatemia caused by renal phosphate wasting with normal or low 1,25-dihydroxyvitamin D concentrations [8]. Low serum phosphate concentration and reduced tubular resorption of phosphate corrected for glomerular filtration rate (TmP/GFR) are characteristic for XLH [9]. Comprehensive diagnoses for XLH need are based on clinical findings, radiographic signs, laboratory tests and genetic analyses.

The recently developed next-generation sequencing (NGS) approach enables parallel sequencing of a large panel of candidate genes with high-efficiency, and has therefore been proved as an efficient tool for the molecular diagnosis of multiple diseases [10]. NGS includes whole exome sequencing (WES), targeted gene capture array sequencing and whole sequencing for mapped choromsomal region. Herein, we report the application of WES to detect the disease causative mutation for a Chinese family showing complicated clinical manifestations with significant intrafamilial diversity.

## Methods

### Study subjects and clinical assessments

**Figure 1:**
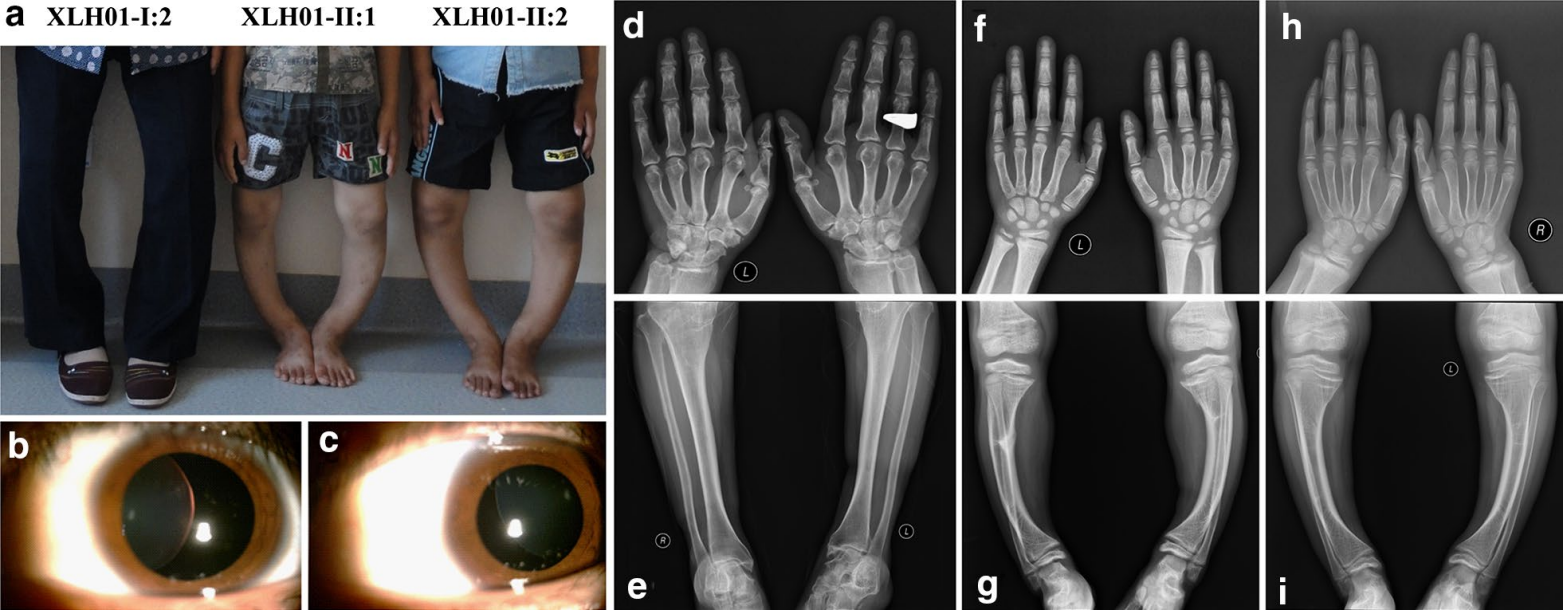
Clinical presentation for family XLH01. **a** Medial bowing in the patients XLH01-I:2, XLH01-II:1 and XLH01-II:2. **b**, **c** Anterior segment photography indicates dislocation of lens toward nasal superior side in both eyes. **d**–**i** Radiographic findings reveal dolichostenomelia in the digits of patient XLH01-II:2 (**h**), but not in patient XLH01-I:2 (**d**) or XLH01-II:1 (**f**). Widening of both proximal and distal tibial metaphysis in patients XLH01-II:1 (**f**, **g**) and XLH01-II:2 (**h**, **i**) are shown. Medial bowing is found in all three patients (**e**, **g**, **i**).

**Figure 2:**
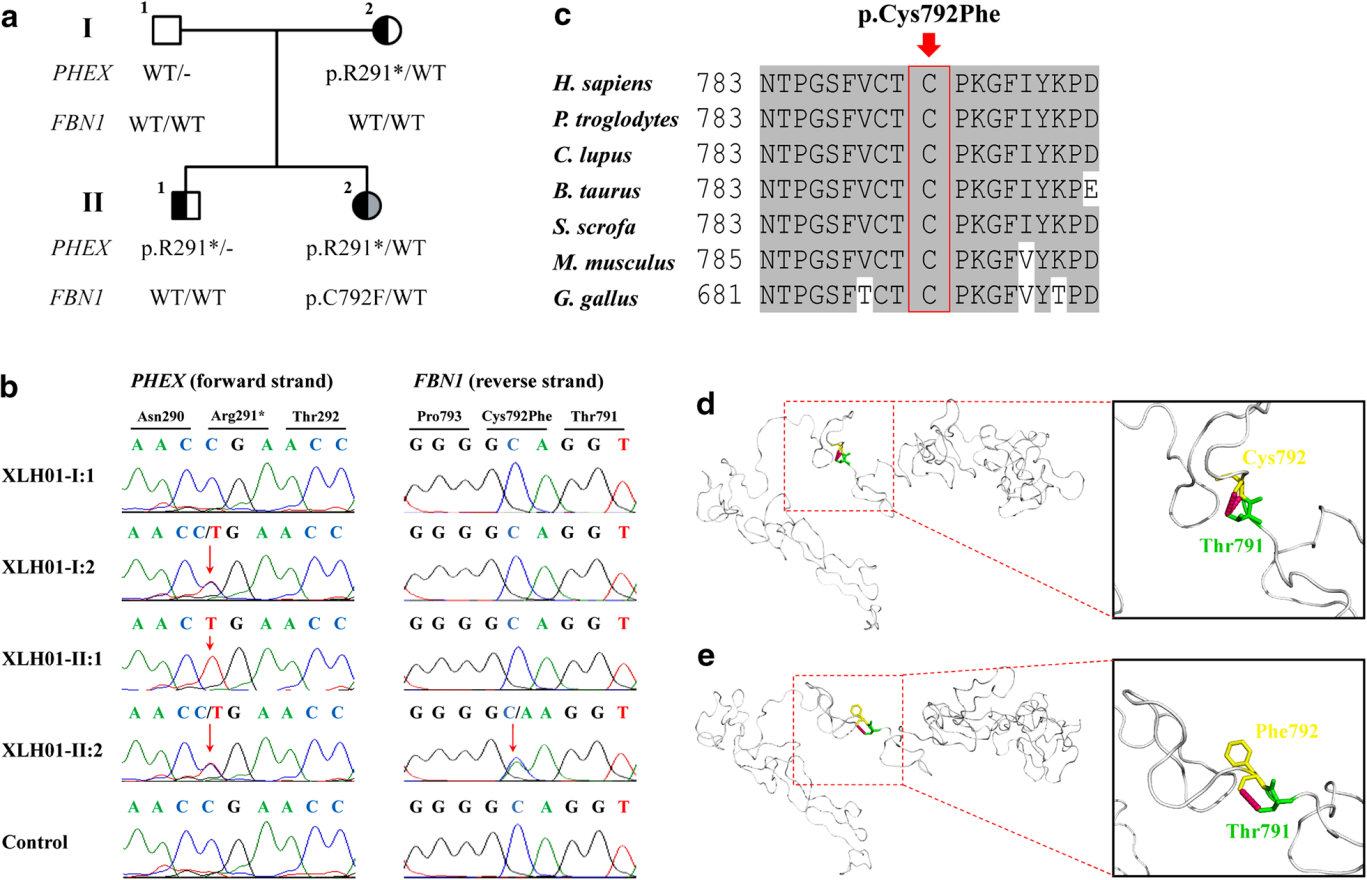
*PHEX* and *FBN1* mutations identified in the current family. **a** Pedigree of the included family. The *PHEX* and *FBN1* genotypes for all included members were annotated. *Black filled*, *grey filled*, and *blank symbols* represent X-linked hypophosphatemia, Marfan syndrome, and unaffected status, respectively. **b** DNA sequencing profiles for the identified disease-causing mutations in the *PHEX* gene (*left panel*) and the *FBN1* gene (*right panel*). **c** Orthologous protein sequence alignment of *PHEX* from seven species. Conserved residues are *shaded*. The mutated residue 792 is *boxed* and indicated. **d**–**e** Structural modeling of the wild type and mutant fibrillin-1. One hydrogen bond in the wide type protein was eliminated due to the substitution from cysteine to phenylalanine at residue 792.

Four participants from family XLH01 of Hui ethnicity, including three patients and an unaffected family member, were recruited from the People’s Hospital of Ningxia Hui Autonomous Region (Figures 1a, 2a). Our study conformed to the Declaration of Helsinki, and was approved and prospectively reviewed by local ethics committee. Written informed contents were obtained from all participants or their legal guardians before their enrollments. Peripheral blood samples were collected from all four participants using 5 mL tubes with ethylene diamine tetraacetic acid (EDTA), and a QIAmp DNA blood kit (Qiagen, Valencia, CA) was used for genomic DNA extraction. Family history was carefully reviewed. Medical and personal history was obtained from each participant. Detailed systemic evaluations and laboratory tests were performed on all included family members, including ophthalmic, radiological, cardiological examinations. Additionally, another 100 healthy controls free of MFS and XLH from the same ethnic group were also recruited with their blood samples collected [11–14].

### Exome sequencing and bioinformatics analysis

Exome sequencing was employed on all three patients of this family, including patients I:2, II:1 and II:2, to identify the disease causative mutation for this family. One μg genomic DNA sample for each patient was sheared into 300–500 bp fragments, ligated with Illumina Y shaped adaptors, purified using the Agencourt AMPure SPRI beads, and amplified through ligation-mediated polymerase chain reaction. The SeqCap EZ Hybridization and Wash kit (Roche NimbleGen, Madison, WI) covering 44.1 megabases (Mb) was then employed for enrichment of over 20,000 genes per the manufacture’s protocols. The post-capture libraries were quantified using Pico green assay and then sequenced on an Illumina Hiseq 2000 machine as indicated previously [15].

Data were then processed as described previously [15]. Annotations of genes and exons for SeqCap EZ Human Exome Library v2.0 were conducted based on the national center of biotechnology information (NCBI) RefSeq project (Jan 2010), the Consensus CDS (CCDS; Sept 2009) and miRBase (v.14, Sept 2009). Sequence reads were aligned to the NCBI human reference genome (NCBI build 37.1) for SNP analysis with SOAP (http://www.soap.genomics.org.cn), and for Indel detection using BWA (http://www.bio-bwa.sourceforge.net/). Coverage and depth calculations were further performed as detailed previously [15]. Detected variants were then filtered against the following databases, including 1000 Genome Project (ftp://ftp.1000genomes.ebi.ac.uk/vol1/ftp), dbSNP135 (http://www.hgdownload.cse.ucsc.edu/goldenPath/hg19/database/snp135.txt.gz.), Exome Variant Server (http://www.evs.gs.washington.edu/EVS/), NIEHS Exome Sequencing database (http://www.evs.gs.washington.edu/niehsExome/), ExAC Browser (http://www.exac.broadinstitute.org/), and an internal control database including 997 exomes [16]. We used a cut-off of minor allele frequency (MAF) 0.1% to reveal variations shared by all three patients and *de novo* variants carried by the proband girl. An additional cut-off of 1% was also set to seek for homozygous and compound heterozygous variants that may help to explain the sole phenotypes of the proband girl.

### Sanger sequencing and in silico analysis

Sanger sequencing was then conducted for mutation verification and prevalence test in 100 unrelated controls using a previously defined protocol [17]. Information of primers was presented in Additional file 1: Table S1. Evolutionary conservation of the mutated amino acids was assessed via Vector NTI Advance 11 (Invitrogen, Grand Island, NY) through alignment of the fibrillin-1 orthologous protein sequences of the following species: *Homo sapiens* (ENSP00000368682), *Pan troglodytes* (ENSPTRP00000037269), *Canis lupus familiaris* (ENSCAFP00000019648), *Bos taurus* (ENSBTAP00000000540), *Sus scrofa* (ENSSSCP00000030440), *Mus musculus* (ENSMUSP00000078863), and *Gallus gallus* (ENSGALP00000026360). SIFT Human Protein DB (http://www.sift.jcvi.org/) and PolyPhen-2 (http://www.genetics.bwh.harvard.edu/pph2/) online softwares were used to detect the potential pathogenic impacts of the mutation. Crystal structures of the wide type and mutant phosphate-regulating neutral endopeptidase were obtained with SWISS-MODEL online server (http://www.swissmodel.expasy.org/). Predicted structures were displayed with PyMol software (version 1.5).

## Results

### Clinical assessments

In June, 2011, the 8-year-old proband (XLH01-II:1) was first referred to our ophthalmic clinic for blurred vision over the past 2 years. She was born at term to a 34-year-old woman after an uneventful full-term pregnancy and delivery. Her best corrected visual acuities (BCVAs) reached 0.1 for the right eye and 0.25 for the left eye. Slit-lamp test revealed dislocation of lens toward nasal superior side and hippus in both eyes (Figure 1b, c). Medical records included an atrioseptopexy at age five for her atrial septal defect (ASD). Other than the repaired ASD presentation, ultrasonic cardiogram (UCG) indicated left-to-right shunt flow through a ventricular septal defect (VSD) with the diameter of 4.50 mm. Slight mitral, tricuspid, and pulmonary regurgitations were also detected. Her calculated Z-score for aortic root was −0.54.

Physical examination on our patient also showed complex skeletal problems. Her height was 117.0 cm [<−2 standard deviation (SD)] and her weight was 30.0 kg (>+1 SD) on admission. Radiographic examinations showed widening of both proximal and distal tibial metaphysis with medial bowing, but dolichostenomelia in her digits were also revealed (Figure 1h). Laboratory analyses indicated hypophosphatemia, elevated serum alkaline phosphatase (ALP) level, and reduced tubular resorption of phosphate corrected for glomerular filtration rate (TmP/GFR) in this patient (Table 1). Serum calcium, intact-parathyroid hormone (iPTH), 25-hydroxyvitamin D3, and 1, 25-dihydroxyvitamin D3 were within the normal range. Her 41-year-old mother (XLH01-I:2) and 10-year-old brother (XLH01-II:2) had no remarkable ophthalmic or cardiac conditions, but showed similar skeletal abnormalities as demonstrated by physical and laboratorial tests with exception of dolichostenomelia in their digits (Figure 1d, g; Table 1). Her brother’s height was 113.0 cm (<−3 SD). Her mother was 145 cm in height and had osteomalacia, severe ostealgia, and difficulties in walking, but no remarkable change in serum ALP level.

**Table 1:** Laboratory analyses for family XLH01 at their initial visit

Indexes	Normal Range	XLH01-I:2	XLH01-II:1	XLH01-II:2
Age (years)	–	41	8	10
Sex	–	Female	Male	Male
TmP/GFR (mg/dL)	2.2–3.6^¶^; 2.9–6.5^†^	0.43	0.85	0.92
Serum ALP (U/L)	40–110	69	337	589
Serum iPTH (pg/mL)	14–72	65.3	43.8	45.4
Serum 25-hydroxyvitamin D3 (nmol/L)	5–200	65	105	95
Serum 1, 25-dihydroxyvitamin D3 (pmol/L)	60–108	75	85	88
Serum calcium (mmol/L)	2.1–2.6^¶^; 2.25–2.8^†^	2.36	2.38	2.38
Serum potassium (mmol/L)	3.5–5.5	0.79	0.91	0.92
Serum sodium (mmol/L)	135–145	140	140	144
Serum chloride (mmol/L)	96–106	102	102	97
Serum phosphonium (mmol/L)	1.0–1.6^¶^; 1.3–1.9^†^	0.79	0.91	0.92
Serum magnesium (mmol/L)	0.7–1.1	1.10	0.84	0.72
Urine amino-acids*	(−)	(−)	(−)	(−)
Urine glucose*	(−)	(−)	(−)	(−)
Urine protein*	(−)	(−)	(−)	(−)

*ALP* Alkaline phosphatase, *iPTH* intact-parathyroid hormone and *TmP/GFR* tubular resorption of phosphate corrected for glomerular filtration rate.

* Qualitative test; ^¶^for adult; ^†^for child; (−) negative.

The proband was thereafter treated with a surgical removal of the dislocated lens with implantation of an artificial intraocular lens and received amblyopia training after surgery. At the 3-year follow-up, her BCVAs improved to 0.4/0.5 (OD/OS). She also had follow-up visits in the cardiology and pediatric departments and was supplemented with elemental phosphorus and calcitriol. Her ALP level was back to normal at 2-year follow-up with no complication.

### Genetic findings

To assist the clinical diagnosis in this unusual family, we performed whole exome sequencing (WES) on patients XLH01-I:2, XLH01-II:1 and XLH01-II:2 (Table 2). The average mean depth of the three tested samples was 33.7X. A total of 24 variants passed the initial filtration process and were confirmed carried by all three patients and absent in the unaffected father (Additional file 1: Table S1). Conversely, a different set of 24 variants with MAF <0.01% in the Chinese population present in the proband but not in the brother or mother were selected to confirm paternity of the father. All 24 variants were observed as heterozygotes in the father (Additional file 1: Table S2), a finding that is not likely to occur in the setting of non-paternity. Among the initial 24 post-filtration variants, a recurrent heterozygous/hemizygous mutation in the *PHEX* gene (c.871C>T [NM_000444]; p.R291* [NP_000435]) was identified in all three affected individuals (Figure 2a, b). This mutation was previously reported in a patient with XLH, and was predicted to generate a premature termination codon at residue 291, which would suspend translation [18]. We therefore believed that this *PHEX* variation was more likely the disease causing mutation for this family than other identified variants. In addition, a *de novo* heterozygous mutation in the *FBN1* gene (c.2375G>T [NM_000138]) was only called in the affected girl (Figure 2a, b). This mutation would cause the amino acid change from the hydrophilic cysteine to the hydrophobic phenylalanine at residue 792 (p.C792F [NP_000129]). Residue Cys792 was predicted to be highly conserved among multiple species (Figure 1c), and both SIFT (score: 0.02; damaging) and PolyPhen-2 (score: 0.997; probably damaging) softwares suggested the deleterious impact of this substitution. Crystal structural modeling of the wide type and mutant fibrillin-1 (residues 584-950) was constructed on the basis of low-density lipoprotein receptor (protein data bank [PDB] ID: 1N7D) with the sequence similarity of 0.38 (Figure 2d, e) [19]. One hydrogen bond between residue 792 and Thr791 was eliminated due to the substitution from cysteine to phenylalanine, which significantly altered its tertiary structure and would further change relevant protein properties.

**Table 2:** Overview of data production

Items	XLH01-I:2	XLH01-II:1	XLH01-II:2
Raw reads	52,834,894	65,462,716	60,937,646
Reads mapped to genome	50,880,007	63,035,145	58,693,151
Mapping rate (%)	96.30	96.29	96.31
Initial number of generated SNVs	178,276	207,524	187,354
Initial number of generated Indels	7,632	8,235	7,815
Mean depth of target region (X)	28.23	37.63	35.12
Coverage of target region (%)	96.76	96.71	96.69
Rate of nucleotide mismatch (%)	0.29	0.26	0.23
Fraction of target covered ≥4 X (%)	93.85	94.83	94.61
Fraction of target covered ≥10 X (%)	75.49	80.56	79.45
Fraction of target covered ≥20 X (%)	57.48	66.88	64.97
Duplication rate (%)	7.92	9.59	8.80
Gender test result	F	M	F

*SNV* Single nucleotide variants and *Indel* insertions and deletions.

## Discussion

MFS and XLH are both systemic diseases but with opposite skeletal changes. MFS and XLH have not been simultaneously reported in any individual before. In our case, the affected girl had a broad spectrum of systemic anomalies, including bilateral ectopia lentis, ASD, VSD, widening of tibial metaphysis with medial bowing, dolichostenomelia in digits, hypophosphatemia, elevated serum ALP level, and reduced TmP/GFR. Similar skeletal and laboratory changes, but except for dolichostenomelia in digits, ocular or cardiac conditions, were also demonstrated by her mother and older brother. The phenotypic complexity and intrafamilial diversity have challenged the original diagnosis for this family, especially for the proband. With the power of WES, we have identified a nonsense mutation *PHEX* p.R291* shared by all three patients, plus a *de novoFBN1* p.C792F exclusively in the proband. The radiological findings, laboratory results, and presence of the nonsense *PHEX* mutation in all 3 patients suggest the diagnosis of XLH for this family [9]. The bilateral ectopia lentis, dolichostenomelia in digits, and the cysteine substitution in *FBN1*, the most common type of *FBN1* mutation that correlates with moderate phenotypes of presumed MFS, revealed in the proband confer an additional diagnosis of MFS to this patient [2]. Another diagnosis of congenital heart disease (CHD) was also finalized to this proband based on the ASD and VSD presentations. Therefore, the clinical presentations and genetic findings confirm the diagnosis of XLH in this family, and MFS combined with XLH and CHD in the 8-year-old proband. To the best of our knowledge, this is the first report of the comprehensive diagnosis of MFS, XLH and CHD in a single patient. Our report also supports the significant role of clinical genetics in the diagnosis of complicated diseases.

Traditional approaches for mutation detection have their limitations, usually resulting into their low diagnostic rate. When compared with traditional methods, NGS has revolutionized the speed and cost for generating large quantities of sequence data [10]. In the present study, mutations were identified via a series of genetic analyses including initial WES, optimized bioinformatics analysis, cosegregation analyses, and mutation screening in control groups [12, 15]. In silico analyses, including the crystal structure modeling tests, also support pathogenicity of identified mutations [13]. With these efforts, our results strongly support that those variants detected are disease-causing mutations rather than rare polymorphisms, and suggests NGS as a promising technology for identifying the novel disease-causing genes associated with monogenic diseases. However, our study also has limitations. WES of the proband alone followed by Sanger sequencing of the variants identified would be a more economic and sufficient strategy for this case.

## Conclusion

In summary, our study not only reports an extremely rare case, but also emphasizes the importance of recently developed approach of WES in the assistance of clinical diagnosis for complicated cases. Genetic counselling and molecular diagnosis would significantly assist clinical diagnoses for such patients with complicated phenotypes, and direct the clinical management.

## Additional file

**Additional file 1: Supplementary Tables.**

## References

[1] von Kodolitsch Y, Robinson PN (2007). Marfan syndrome: an update of genetics, medical and surgical management. Heart.

[2] Dietz HC (1993) Marfan Syndrome. GeneReviews. University of Washington, Seattle. http://www.ncbi.nlm.nih.gov/books/NBK1335/20301510

[3] Zhao F, Pan X, Zhao K, Zhao C (2013). Two novel mutations of fibrillin-1 gene correlate with different phenotypes of Marfan syndrome in Chinese families. Mol Vis.

[4] Mizuguchi T, Collod-Beroud G, Akiyama T, Abifadel M, Harada N, Morisaki T (2004). Heterozygous TGFBR2 mutations in Marfan syndrome. Nat Genet.

[5] White KE, Evans WE, O’Riordan JL, Speer MC, Econs MJ, Lorenz-Depiereux B (2000). Autosomal dominant hypophosphataemic rickets is associated with mutations in FGF23. Nat Genet.

[6] Lorenz-Depiereux B, Bastepe M, Benet-Pages A, Amyere M, Wagenstaller J, Muller-Barth U (2006). DMP1 mutations in autosomal recessive hypophosphatemia implicate a bone matrix protein in the regulation of phosphate homeostasis. Nat Genet.

[7] Levy-Litan V, Hershkovitz E, Avizov L, Leventhal N, Bercovich D, Chalifa-Caspi V (2010). Autosomal-recessive hypophosphatemic rickets is associated with an inactivation mutation in the ENPP1 gene. Am J Hum Genet.

[8] Cheon CK, Lee HS, Kim SY, Kwak MJ, Kim GH, Yoo HW (2014). A novel de novo mutation within PHEX gene in a young girl with hypophosphatemic rickets and review of literature. Ann Pediatr Endocrinol Metab.

[9] Ruppe MD (1993) X-Linked Hypophosphatemia. GeneReviews. University of Washington, Seattle. http://www.ncbi.nlm.nih.gov/books/NBK83985/22319799

[10] Shendure J, Ji H (2008). Next-generation DNA sequencing. Nat Biotechnol.

[11] Rong W, Chen X, Zhao K, Liu Y, Liu X, Ha S (2014). Novel and recurrent MYO7A mutations in Usher syndrome type 1 and type 2. PLoS One.

[12] Chen X, Liu Y, Sheng X, Tam PO, Zhao K, Rong W (2014). PRPF4 mutations cause autosomal dominant retinitis pigmentosa. Hum Mol Genet.

[13] Chen X, Sheng X, Liu X, Li H, Liu Y, Rong W (2014). Targeted next-generation sequencing reveals novel USH2A mutations associated with diverse disease phenotypes: implications for clinical and molecular diagnosis. PLoS One.

[14] Pan X, Chen X, Liu X, Gao X, Kang X, Xu Q (2014). Mutation analysis of pre-mRNA splicing genes in Chinese families with retinitis pigmentosa. Mol Vis.

[15] Chen X, Zhao K, Sheng X, Li Y, Gao X, Zhang X (2013). Targeted sequencing of 179 genes associated with hereditary retinal dystrophies and 10 candidate genes identifies novel and known mutations in patients with various retinal diseases. Invest Ophthalmol Vis Sci.

[16] Fu Q, Wang F, Wang H, Xu F, Zaneveld JE, Ren H (2013). Next-generation sequencing-based molecular diagnosis of a Chinese patient cohort with autosomal recessive retinitis pigmentosa. Invest Ophthalmol Vis Sci.

[17] Zhao C, Lu S, Zhou X, Zhang X, Zhao K, Larsson C (2006). A novel locus (RP33) for autosomal dominant retinitis pigmentosa mapping to chromosomal region 2cen-q12.1. Hum Genet.

[18] Holm IA, Huang X, Kunkel LM (1997). Mutational analysis of the PEX gene in patients with X-linked hypophosphatemic rickets. Am J Hum Genet.

[19] Rudenko G, Henry L, Henderson K, Ichtchenko K, Brown MS, Goldstein JL (2002). Structure of the LDL receptor extracellular domain at endosomal pH. Science.

