# Supplementary material for: Whole exome sequencing confirms the clinical diagnosis of Marfan syndrome combined with X-linked hypophosphatemia
Source: J Transl Med. 2015 Jun 4;13:179. doi: 10.1186/s12967-015-0534-9 (PMC4455986; doi:10.1186/s12967-015-0534-9)
Supplement: Supplementary file 1 — Additional file 1: Supplementary Tables. [file 12967_2015_534_MOESM1_ESM.docx]

**Supplementary materials**

Supplementary materials included two Tables (**Table S1** and **S2**).

| **Table S1 Missense variants shared by affected individuals post-filtration.** | | | | | | | |
| --- | --- | --- | --- | --- | --- | --- | --- |
| **Chromosome** | **Gene** | **Variations (NCBI36/hg18)** | | | **Variant status** | | |
|  |  | **Location** | **Nucleotide change** | **Amino acid change** | **XLH01-I:2** | **XLH01-II:1** | **XLH01-II:2** |
| Chr 1 | *PADI3* | 17482058 | c.1892T>C | p.Ile631Thr | Het. | Het. | Het. |
| Chr 1 | *GPR3* | 27593788 | c.899G>C | p.Arg300Pro | Het. | Het. | Het. |
| Chr 1 | *ATG4C* | 63043431 | c.77G>T | p.Ser26Ile | Het. | Het. | Het. |
| Chr 1 | *IL12RB2* | 67618368 | c.1829G>A | p.Gly610Glu | Het. | Het. | Het. |
| Chr 2 | *ADCY3* | 24899584 | c.2881T>G | p.Ser961Ala | Het. | Het. | Het. |
| Chr 2 | *THADA* | 43637143 | c.2519C>G | p.Thr840Ser | Het. | Het. | Het. |
| Chr 2 | *LRP2* | 1.7E+08 | c.9707G>A | p.Arg3236Gln | Het. | Het. | Het. |
| Chr 5 | *AFF4* | 1.32E+08 | c.2567A>G | p.Lys856Arg | Het. | Het. | Het. |
| Chr 5 | *ANXA6* | 1.5E+08 | c.1120A>G | p.Lys374Glu | Het. | Het. | Het. |
| Chr 10 | *GTPBP4* | 1051769 | c.1685C>G | p.Ser562Cys | Het. | Het. | Het. |
| Chr 11 | *AMOTL1* | 94194499 | c.1277C>T | p.Pro426Leu | Het. | Het. | Het. |
| Chr 11 | *C11orf53* | 1.11E+08 | c.682G>T | p.Gly228Trp | Het. | Het. | Het. |
| Chr 11 | *BUD13* | 1.16E+08 | c.670G>T | p.Asp224Tyr | Het. | Het. | Het. |
| Chr 11 | *APLP2* | 1.3E+08 | c.1549G>T | p.Asp517Tyr | Het. | Het. | Het. |
| Chr 12 | *SIRT4* | 1.3E+08 | c.530C>T | p.Pro177Leu | Het. | Het. | Het. |
| Chr 13 | *SLITRK1* | 83351884 | c.1760C>T | p.Pro587Leu | Het. | Het. | Het. |
| Chr 14 | *SYNE2* | 63527584 | c.2644A>G | p.Lys882Glu | Het. | Het. | Het. |
| Chr 15 | *HERC1* | 61753657 | c.7783A>G | p.Met2595Val | Het. | Het. | Het. |
| Chr 17 | *MYH3* | 10482287 | c.3527G>A | p.Arg1176His | Het. | Het. | Het. |
| Chr 17 | *IFI35* | 38419073 | c.436C>T | p.Leu146Phe | Het. | Het. | Het. |
| Chr 18 | *DSG4* | 27220758 | c.194C>T | p.Ser65Leu | Het. | Het. | Het. |
| Chr 21 | *GART* | 33822750 | c.842A>G | p.Lys281Arg | Het. | Het. | Het. |
| Chr X | *EGFL6* | 13555115 | c.1350A>C | p.Lys450Asn | Het. | Hem. | Het. |
| **Abbreviations:** Het: heterozygous; Hem: hemizygous. | | | | | | | |

| **Table S2 Rare variants (MAF < 0.1%) observed in proband and genotyped in father to confirm paternity.** | | | | | | | | |
| --- | --- | --- | --- | --- | --- | --- | --- | --- |
| **Chromosome** | **Gene** | **Variations (NCBI36/hg18)** | | | **Variant status** | | | |
|  |  | **Location** | **Nucleotide change** | **Amino acid change** | **XLH01-I:1** | **XLH01-I:2** | **XLH01-II:1** | **XLH01-II:2** |
| Chr 1 | *BAT2D1* | 169768122 | c.1265G>A | p.Arg422Gln | Het. | WT | WT | Het. |
| Chr 2 | *CCDC74B* | 130614337 | c.761C>T | p.Ala253Val | Het. | WT | WT | Het. |
| Chr 3 | *TOPBP1* | 134830141 | c.2647G>T | p.Val883Phe | Het. | WT | WT | Het. |
| Chr 4 | *LYAR* | 4334475 | c.173A>G | p.Tyr58Cys | Het. | WT | WT | Het. |
| Chr 5 | *AGXT2* | 35075330 | c.218A>G | p.His218Arg | Het. | WT | WT | Het. |
| Chr 6 | *RNGTT* | 89380776 | c.1564A>G | p.Asn522Asp | Het. | WT | WT | Het. |
| Chr 8 | *DOCK5* | 25280367 | c.3188C>T | p.Thr1063Ile | Het. | WT | WT | Het. |
| Chr 9 | *IL11RA* | 34650537 | c.1109C>T | p.Ala370Val | Het. | WT | WT | Het. |
| Chr 10 | *BLOC1S2* | 102030734 | c.239T>C | p.Met80Thr | Het. | WT | WT | Het. |
| Chr 11 | *CRTAM* | 122238457 | c.728G>A | p.Ser243Asn | Het. | WT | WT | Het. |
| Chr 12 | *TSPAN9* | 3258010 | c.226A>C | p.Ile76Leu | Het. | WT | WT | Het. |
| Chr 13 | *AKAP11* | 41773589 | c.2707C>T | p.Arg903Cys | Het. | WT | WT | Het. |
| Chr 14 | *GALNTL1* | 68861839 | c.467A>G | p.Gln156Arg | Het. | WT | WT | Het. |
| Chr 15 | *HERC2* | 26146993 | c.5430G>T | p.Met1810Ile | Het. | WT | WT | Het. |
| Chr 15 | *GCNT3* | 57698469 | c.740A>G | p.Asn247Ser | Het. | WT | WT | Het. |
| Chr 15 | *PSTPIP1* | 75116542 | c.1221C>A | p.Phe407Leu | Het. | WT | WT | Het. |
| Chr 15 | *PDE8A* | 83461992 | c.1514C>G | p.Ser505Cys | Het. | WT | WT | Het. |
| Chr 16 | *VWA3A* | 22037743 | c.431T>C | p.Met144Thr | Het. | WT | WT | Het. |
| Chr 17 | *MINK1* | 4740550 | c.3308C>T | p.Thr1103Met | Het. | WT | WT | Het. |
| Chr 18 | *DSG3* | 27309995 | c.2774C>T | p.Pro925Leu | Het. | WT | WT | Het. |
| Chr 19 | *PSG11* | 48220772 | c.341G>A | p.Arg114Gln | Het. | WT | WT | Het. |
| Chr 21 | *PWP2* | 44365005 | c.1403C>G | p.Ala468Gly | Het. | WT | WT | Het. |
| Chr 22 | *RFPL3* | 31086358 | c.493G>A | p.Val165Met | Het. | WT | WT | Het. |
| Chr X | *XPNPEP2* | 128705685 | c.191A>G | p.Gln64Arg | Het. | WT | WT | Hem. |
| **Abbreviations:** Het.: heterozygous; Hem.: hemizygous; WT: wild type. | | | | | | | | |
